# Role of adiponectin and leptin on body development in infants during the first year of life

**DOI:** 10.1186/1824-7288-36-26

**Published:** 2010-03-18

**Authors:** Elena Bozzola, Cristina Meazza, Marica Arvigo, Paola Travaglino, Sara Pagani, Mauro Stronati, Antonella Gasparoni, Carolina Bianco, Mauro Bozzola

**Affiliations:** 1 Paediatrics Department, University of Pavia, Fondazione IRCCS Policlinico San Matteo, Piazzale Golgi 2, Pavia, Italy; 2 Department of Endocrinological and Metabolic Sciences, University of Genova, Genova, Italy; 3 Neonatal Intensive Care, IRCCS San Matteo, Pavia, Italy; 4 Neonatal Intensive Care, Spedali Civili, Brescia, Italy

## Abstract

**Background:**

The control of growth and nutritional status in the foetus and neonate is a complex mechanism, in which also hormones produced by adipose tissue, such as adiponectin and leptin are involved. The aim of this study was to evaluate levels of adiponectin, leptin and insulin in appropriate (AGA) and small for gestational age (SGA) children during the 1^st ^year of life and to correlate these with auxological parameters.

**Methods:**

In 33 AGA and 29 SGA infants, weight, length, head circumference, glucose, insulin, adiponectin and leptin levels were evaluated at the second day of life, and at one, six and twelve months, during which a portion of SGA could show catch-up growth (rapid growth in infants born small for their gestational age).

**Results:**

Both total and isoform adiponectin levels were comparable between AGA and SGA infants at birth and until age one year. These levels significantly increased from birth to the first month of life and then decreased to lower values at 1 year of age in all subjects. Circulating leptin concentrations were higher in AGA (2.1 ± 4.1 ng/ml) than in SGA neonates (0.88 ± 1.03 ng/ml, p < 0.05) at birth, then similar at the 1^st ^and the 6^th ^month of age, but they increased in SGA from six months to one year, when they showed catch-up growth. Circulating insulin levels were not statistically different in AGA and SGA neonates at any study time point. Insulin levels in both AGA and SGA infants increased over the study period, and were significantly lower at birth compared to one, six and 12 months of age.

**Conclusions:**

During the first year of life, in both AGA and SGA infants a progressive decrease in adiponectin levels was observed, while a difference in leptin values was correlated with the nutritional status.

## Background

Epidemiological studies have shown an association between a reduced size at birth and increased long-term risk for obesity, insulin resistance, type 2 diabetes, hypertension and cardiovascular disease in adulthood [1-3]. Low birth weight has been associated with hyperinsulinaemia in both children [4,5] and adults [6], suggesting that this metabolic abnormality may be the link between reduced prenatal growth and certain endocrinological diseases in adulthood. In particular, the transition from a relatively low birth weight to larger post-natal body size is associated with an increased risk for insulin resistance [7-9]. It, therefore, seems that small birth size, and particularly low birth weight are factors that greatly increase the risk of developing endocrine and metabolic diseases in adulthood [9].

A role for adipose tissue in the pathophysiology of insulin resistance has been postulated in several studies [10]. It is currently known to secrete a large number of factors with diverse functions, such as free fatty acids, with well described physiological and pathophysiological effects on glucose homeostasis [11], and proteins, termed adipocytokines, that act in an autocrine, paracrine, or endocrine fashion to control various metabolic functions. Some of these adipocytokines, such as adiponectin, leptin, tumour necrosis factor-α (TNF-α) and interleukin-6 (IL-6), may act locally or distally to alter insulin sensitivity in insulin-targeted organs such as muscle and the liver or may act through neuroendocrine, autonomic or immune pathways [12]. Adiponectin and leptin are considered the most important hormones related to adipose depots in modulating metabolism and energy homeostasis [12].

In adult humans, plasma adiponectin is an important insulin sensitizer, as its circulating level is inversely related to the body mass index and to measures of insulin resistance [13]. Since foetal growth is to a great extent controlled by the actions of insulin, adiponectin could also be expected to have significant effects on foetal growth and development. In the circulation, adiponectin exists as low-, medium- and high molecular weight complexes that appear to elicit different effects on target tissues [14]. In particular, the high molecular weight isoform has been linked with insulin-sensitizing activity [15].

Circulating leptin concentrations, mainly produced by white adipose tissue, correlate with the development of foetal adipose tissue and later with the body mass index [16]. The role of leptin among the complex network of factors controlling foetal growth is incompletely understood, even though recent studies have demonstrated a strong correlation between leptin in cord blood and foetal birth weight [17,18].

The aim of the present study was to evaluate serum levels of leptin, adiponectin and insulin in infants born appropriate for gestational age (AGA) and small for gestational age (SGA) during the first year of life and to correlate these changes with auxological parameters.

## Methods

Thirty-three AGA (birth weight > -2 SD according to gestational age and gender) [19] infants (21 males and 12 females) and 29 SGA (birth weight < -2 SD according to gestational age and gender) infants (19 males and 10 females), participated in the study. According to gestational age at delivery, all the neonates were full-term (38 ± 1 weeks of gestation). Gestational age at delivery was calculated according to the last menstrual period and confirmed by ultrasound examination during the first trimester or early second trimester. The mothers of the neonates enrolled confirmed the normal course of the pregnancy, without any complications or administration of drugs. Thirty-five neonates (17 SGA and 18 AGA) were born by eutocic birth, while 27 (12 SGA and 15 AGA) were delivered by caesarean section.

Neonates were randomly recruited at birth from the Neonatal Units of the Spedali Civili Hospital of Brescia and the San Matteo Hospital of Pavia, Italy.

Subjects with evidence of malformations or genetic disorders were excluded from the study. Mid-parental height, used as an indicator of the genetic growth potential, was similar in AGA (mean ± standard deviation: 0.31 ± 0.82 SDS, standard deviation score) and SGA groups (-0.12 ± 0.85 SDS).

Among SGA infants, 16 were symmetrical and 13 asymmetrical. We defined "symmetrical SGA" neonates as having both birth length and weight less than 2 SDS corrected for gestational age and "asymmetrical SGA" neonates with birth weight only less than -2 SDS corrected for gestational age.

The infants had similar diets, during the first year of life, they received only milk until the fourth month, then one solid meal was introduced at the sixth month and two solid meals at the eighth month.

In all infants, weight, length and head circumference were evaluated at the second day of life, and at one, six and 12 months by a trained paediatrician, and converted to SDS to adjust for age and sex [20,21]. The body mass index (BMI) was used as a measurement of relative adiposity and was calculated according to the formula: weight (Kg)/length^2 ^(m^2^) [21].

At regularly scheduled control visit, a blood sample was obtained from each neonate (in the fasting state) for the determination of glucose, insulin, adiponectin and leptin. In order to avoid possible confounders in assessing hormone levels at birth, we took blood samples from the neonates on the 2^nd ^day of life and did not use cord blood in which hormone levels may derive from the maternal circulation or from other maternal tissues, such as the placenta [22,23].

The study protocol was approved by the local Ethics Committee. The study's purpose was fully explained to each pregnant woman and written informed consent was obtained before enrolment.

Glucose was measured immediately using a commercial glucometer (One touch Ultra, Lifescan, Johnson Johnson company). Blood samples were then centrifuged and serum was frozen at -20°C until the measurements were performed.

Serum adiponectin and leptin concentrations were measured by a commercially available ELISA assay (B-Bridge International, Inc, Sunnyvale CA, USA and R&D Systems, Minneapolis MN, USA, respectively). The minimum detectable concentration for adiponectin was 375 pg/ml and the intra- and inter-assay coefficient of variation ranges were 3.2-7.3% and 4.6-5.8% for a quality control range of 0.7-2.9 ng/ml, respectively. The minimum detectable concentration for leptin was 15.6 pg/ml and the intra- and inter-assay coefficient of variation ranges were 3.2-3.3% and 3.5-5.4% for a quality control range of 65-600 ng/ml, respectively.

Serum insulin levels were measured by an automatic chemiluminescent assay ADVIA centaur IRI (Bayer Diagnostics Europe, Dublin, Ireland). The minimum concentration detectable by this assay is 0.1 μU/ml. The intra- and inter-assay coefficients of variation were 2.1-5.0% and 4.2-6.3% for a quality control range of 0.6-4.0 μU/ml, respectively.

Insulin resistance was estimated with fasting insulin and glucose levels using the homeostasis model assessment insulin resistance index (HOMA-IR), using the following formula: fasting serum insulin (μU/ml) × fasting plasma glucose (mmol/L)/22.5.

Although the HOMA-IR measure is less accurate than the euglycemic clamp method used in large epidemiological studies, it is a reasonable alternative to the complicated clamp method that requires continuous intravenous administration of insulin and glucose for 3 h to calculate insulin sensitivity [24]. A HOMA-IR value > 3 was chosen as an indicator of reduced insulin sensitivity [25].

Even if the gold standard techniques for measuring insulin sensitivity in children are Bergman's minimal model and the hyperinsulinaemic-euglycemic clamp [26,27], the evaluation of insulin sensitivity starting from basal insulin levels has been validated in non-diabetic children as well as in children of one year of age [28-30] and the use of HOMA-IR has been reported in previous paediatric studies [31,32].

Samples of 250 μl of pooled serum were gel-filtered on HyPrep 16/60 Sephacryl-S-200 High Resolution columns equilibrated with 0.05 M NaH_2_PO_4_/0.15 M NaCl/0.02% NaN_3 _elution buffer. One minute fractions, eluted with 0.8 ml/min flow rate, were analysed for adiponectin content with the ELISA assay.

Data were analysed using the statistical analysis software package Statistica 7.0 (Stasoft Inc). Descriptive statistics were calculated and reported as the mean and standard deviation. Non parametric tests were used for statistical analysis. The Mann-Whitney U test for unpaired samples was used to compare anthropometrical and laboratory parameters between SGA and AGA infants, at each time point of the study. The non-parametric Wilcoxon test for paired samples was used to compare anthropometrical and laboratory parameters at different times. Correlations were analysed using the Spearman rank correlation test. A p value < 0.05 was considered statistically significant.

## Results

### Auxology

As shown in table 1, symmetrical SGA infants weighted less and were shorter at birth, at one and 12 months of life compared to AGA infants. On the contrary, asymmetrical SGA infants showed a lower BMI only at birth and at one month compared to AGA infants. Furthermore, head circumferences were smaller in both symmetrical and asymmetrical SGA infants from birth to six months of age but they became comparable to those of AGA infants at one year. Symmetrical SGA infants were, by definition, significantly shorter (p < 0.05) than asymmetrical SGA infants at birth and also at 1, 6 and 12 months of age.

**Table 1:** Anthropometrical measurements of AGA, symmetrical and asymmetrical SGA at birth, 1, 6 and 12 months of life.

AGA	Birth	1 month	6 months	12 months
Length (SDS)	0.31 ± 0.78#	0.39 ± 0.87#	-0.15 ± 0.90	-0.40 ± 0.95
Weight (SDS)	-0.23 ± 0.66#	-0.24 ± 1.22	0.26 ± 0.98	0.19 ± 1.05
BMI (SDS)	-0.06 ± 0.84§	0.70 ± 0.86	0.77 ± 1.14	0.38 ± 1.03
Head circumference (SDS)	0.49 ± 0.76#	0.28 ± 1.05#	0.61 ± 1.14#	-0.70 ± 0.69

**Symmetrical SGA**				

Length (SDS)	-1.82 ± 0.85*	-1.21 ± 1.29*	-1.47 ± 0.81*	-1.85 ± 0.54*
Weight (SDS)	-2.45 ± 0.75*	-2.52 ± 1.97*#	-1.43 ± 1.54*#	-1.93 ± 1.62*
BMI (SDS)	-1.53 ± 0.60*§	-0.61 ± 1.19*	0.33 ± 1.19	-0.27 ± 1.02*
Head circumference (SDS)	-1.01 ± 0.88*	-0.75 ± 1.01*	-0.47 ± 0.76*	-1.25 ± 0.77

**Asymmetrical SGA**				

Length (SDS)	-0.29 ± 0.62^	-0.10 ± 0.69^#	-0.47 ± 0.57^	-0.70 ± 0.61^
Weight (SDS)	-1.84 ± 0.27*#§	-1.25 ± 0.84*	-0.19 ± 1.38	-0.73 ± 1.40
BMI (SDS)	-1.80 ± 0.49*#§	-0.54 ± 1.09*	0.62 ± 1.71#	0.22 ± 1.54
Head circumference (SDS)	-0.86 ± 0.91*	-0.65 ± 1.24*	-0.65 ± 1.35*	-1.31 ± 1.24

* p < 0.05 versus AGA at corresponding time (Mann-Whitney U test)

^ p < 0.05 versus symmetrical SGA at corresponding time (Mann-Whitney U test)

# p < 0.01 versus 12 months with respect to the same group (Wilcoxon test)

§ p < 0.001 versus 1 month with respect to the same group (Wilcoxon test)

Data are shown as mean ± standard deviation.

### Circulating levels of adiponectin

**Figure 1:**
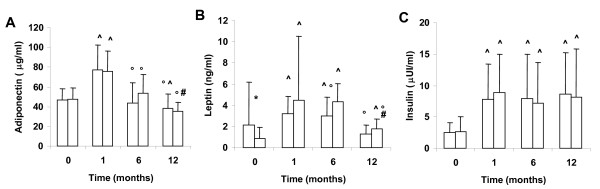
**Circulating levels of adiponectin, leptin and insulin**. Circulating levels of adiponectin (A), leptin (B) and insulin (C) in AGA (white bars) and SGA (shaded bars) infants at birth and after 1, 6 and 12 months of age. Data are represented as mean+standard deviation. * p < 0.05 AGA versus SGA (Mann-Whitney U test). ^ p < 0.05 AGA or SGA with respect to values at birth (Wilcoxon test). ° p < 0.05 AGA or SGA with respect to values at 1 month (Wilcoxon test). # p < 0.05 SGA with respect to values at 6 months (Wilcoxon test).

As shown in figure 1A, both AGA and SGA fasting adiponectin levels showed statistically significant changes over time (p < 0.0005 by Wilcoxon test). In particular, they significantly increased from birth to the first month of life and then decreased to lower values at one year of age in all subjects. Adiponectin levels in AGA and SGA subjects were comparable at all study time points.

**Figure 2:**
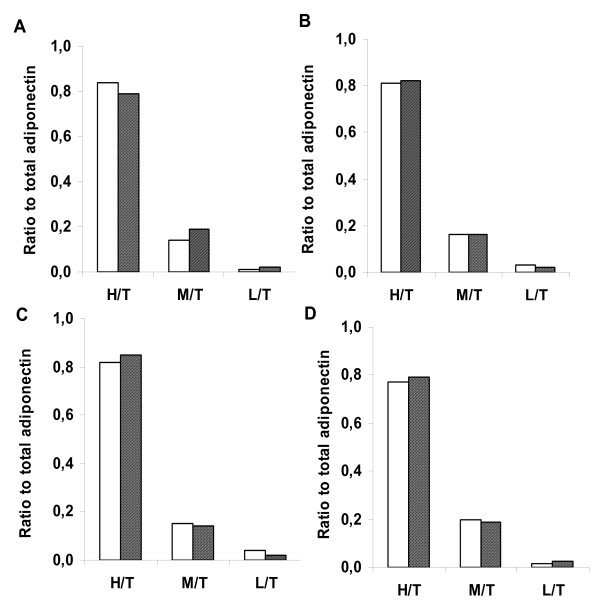
**Fractional ratios to total adiponectin**. Fractional ratios to total adiponectin in AGA (white bars) and SGA (shaded bars) infants at birth (panel A), after 1 (panel B), 6 (panel C) and 12 months (panel D) of age. T = total adiponectin. H = high molecular weight. M = medium molecular weight. L = low molecular weight.

In order to investigate whether SGA and AGA subjects show a different distribution of the isomers for adiponectin, we fractioned adiponectin pooled serum samples into the three major molecular fractions, using a validated fast protein liquid chromatography assay. As shown in figure 2, no differences in the fractional adiponectin levels relative to total adiponectin were found between AGA and SGA groups at different time points.

No statistically significant correlations were found between adiponectin levels and auxological parameters at any time point, in any neonate.

No statistically significant difference in adiponectin levels was shown between symmetrical and asymmetrical SGA infants at birth and during the first year of life (table 2). A significant increase in adiponectin values was observed at the first month of age in both groups of subjects and then a statistically significant decrease up to one year of life. Only at six months of age did asymmetric subjects show higher levels of adiponectin than AGA subjects.

**Table 2:** Circulating levels of adiponectin, leptin and insulin in AGA, symmetrical and asymmetrical SGA at birth, 1, 6 and 12 months of life.

AGA	Birth	1 month	6 months	12 months
Adiponectin (μg/ml)	46.7 ± 11.5#*	77.32 ± 24.95#	43.31 ± 20.94	38.56 ± 13.86
Leptin (ng/ml)	2.12 ± 4.08*	3.18 ± 1.64#	2.97 ± 1.76	1.31 ± 0.83
Insulin (μUI/ml)	2.53 ± 1.58*#	7.75 ± 5.61	7.94 ± 7.06	8.66 ± 6.52

**SGA**				

Adiponectin (μg/ml)	47.74 ± 11.38	75.71 ± 20.75	53.75 ± 18.71	35.51 ± 8.76

Leptin (ng/ml)	0.88 ± 1.04§	4.49 ± 5.99	4.36 ± 1.69§	1.74 ± 0.96

Insulin (μUI/ml)	8.92 ± 6.05	8.92 ± 6.05	7.17 ± 6.44	8.13 ± 7.67

**Symmetrical SGA**				

Adiponectin (μg/ml)	49.03 ± 9.93*	79.78 ± 25.29#	44.42 ± 15.75	33.68 ± 9.17
Leptin (ng/ml)	0.62 ± 0.37*	6.58 ± 8.06	4.67 ± 1.34	2.17 ± 1.29
Insulin (μUI/ml)	2.07 ± 1.97*	8.96 ± 5.55	6.83 ± 3.22	10.0 ± 8.8

**Asymmetrical SGA**				

Adiponectin (μg/ml)	46.73 ± 12.89*	71.64 ± 15.42#	64.65 ± 16.68§	36.82 ± 8.93
Leptin (ng/ml)	1.1 ± 1.4	2.4 ± 1.6	4.0 ± 2.1#	1.4 ± 0.56
Insulin (μUI/ml)	3.17 ± 2.67*	8.89 ± 6.77	7.51 ± 8.91	5.43 ± 3.37

§ p < 0.05 versus AGA at corresponding time (Mann-Whitney U test)

# p < 0.01 versus 12 months of the same group (Wilcoxon test)

* p < 0.001 versus 1 month of the same group (Wilcoxon test)

### Circulating levels of leptin

As shown in figure 1B, leptin levels significantly changed over time both in AGA and SGA infants (p < 0.005 by Wilcoxon test). In fact, leptin levels significantly increased from birth to the sixth month in all the neonates and at one year there was a trend to return to birth levels; although at this time, in SGA subjects, leptin levels were significantly higher than at birth. Circulating leptin concentrations at birth were significantly higher in AGA neonates than in SGA infants. At the first, 6^th ^and 12^th ^month of age, leptin returned to a comparable level between the two groups, even if at the latter time point SGA levels were slightly higher than AGA values, the difference was not significant.

A comparison of leptin levels for asymmetrical and symmetrical SGA infants, did not reveal any statistically significant difference at any time during the study (table 2). In both groups, the levels were low at birth and significantly increased until six months.

Leptin concentrations at birth were positively correlated with the BMI (r = 0.363, p < 0.05), birth head circumference (r = 0.434, p < 0.05) and insulin levels (r = 0.448, p < 0.05) in all the infants. After birth, a positive correlation between leptin levels and BMI at six months was found (R = 0.406; p < 0.05) in all neonates and also for AGA and SGA subjects separately.

### Circulating levels of insulin

As shown in figure 1C, insulin levels in both AGA and SGA infants increased over the study period (p < 0.05 by Wilcoxon test); they were significantly lower (p < 0.05) at birth compared to the time points one, six and 12 months of age. SGA circulating insulin levels at birth were lower than AGA levels, although the difference was not statistically significant. At 1, 6 and 12 months, insulin concentrations were comparable between the two groups.

Insulin concentrations for symmetrical and asymmetrical SGA infants were not statistically different at any study time point (table 2), although in asymmetrical subjects they tended to be lower than in symmetrical infants after the first month of age. In both groups, insulin levels were low at birth and tended to increase after the first month of life.

Insulin concentrations at one year of age positively correlated with the BMI (r = 0.408, p < 0.05) and head circumference (r = 0.436, p < 0.05) in all the infants. No other correlations were found at the other time points.

In order to evaluate insulin resistance in AGA and SGA subjects during the first year of life, we calculated the HOMA-IR index. Like insulin levels, the HOMA-IR index was comparable between AGA and SGA infants at birth and at all study time points (data not shown). The HOMA-IR index was lower at birth in both groups (p < 0.01) and then increased from the first month to 12 months of age, remaining in the normal range [25].

The HOMA-IR index was also comparable between asymmetric and symmetric SGA infants at all time points.

To further verify the relationship between changes in height SDS and BMI SDS with those of adiponectin, leptin and insulin, we calculated the delta between birth and the first year of life for each parameter and the relationship between each parameter pair (data not shown). We did not observe any significant correlations.

## Discussion

This study presents longitudinal data, from birth to one year of age, on anthropometrical development and serum adiponectin, leptin and insulin levels in a cohort of AGA and SGA infants, including both symmetrical and asymmetrical neonates.

To the best of our knowledge, adiponectin and leptin studies at birth have always been performed on cord blood, which is not considered the ideal material for evaluating these growth stimulating-factors. As we previously indicated, cord blood may present confounding factors [22-25,33]. Our study is the first to report actual circulating levels at birth.

The relationship between adiponectin and foetal growth has been poorly investigated. Some authors have found a positive association between cord blood adiponectin levels and birth-weight [33,34], while others have not [35]. In our study, no significant difference in adiponectin levels was shown between AGA and SGA infants at birth, although SGA neonates weighed significantly less than AGA infants. This result is in accordance with the literature, particularly with the Kamoda et al. study [36].

Furthermore, adiponectin levels do not correlate with birth weight and the physiological relationship with adiposity, observed in adults, is absent at birth.

We found that adiponectin levels in both AGA and SGA neonates were higher than those found in healthy adults in other studies [35]. This is probably due to a lack of negative feedback on adiponectin production resulting from a lack of adipocyte hypertrophy, a low percentage of body fat or a different distribution of fat depots in newborns [37]. The analysis of adipose tissue histology in newborns demonstrated the presence of small cells, that do not contain fat, and larger cells, that contain fat but are small in diameter compared with adult fat cells [37]. These cells are responsible for the increased adiponectin synthesis in neonates. The fall in adiponectin levels at one year of life may be a consequence of the increase in adiposity; as in older children, where adiponectin levels negatively correlate with the percentage of body fat. It has also been reported that low plasma adiponectin concentrations are closely related to hyperinsulinaemia in children [38,39]. The lower levels of adiponectin in children at 12 months of age could therefore also be related to the increased concentration of insulin at this time. At one year of age, no statistically significant difference is present in adiponectin levels between AGA and SGA infants, indicating that the long-term risk of diabetes, which has been reported to be associated with low adiponectin levels in prepubertal children [40], is the same in the two groups at this time. In fact, a recent study suggests that the negative correlation between high molecular weight (HMW) adiponectinaemia and fasting insulin levels emerges between two and six years of age and only during this period could a condition of insulin resistance be evidenced [41].

We have also evaluated adiponectin fractions with different molecular weights in SGA and AGA neonates, in order to verify if there was a peculiar distribution of adiponectin isomers. We found a similar chromatographic pattern of adiponectin fractions in AGA and SGA subjects, during the first year of life, confirming the absence of modifications in total circulating adiponectin. Furthermore, in other recent studies by Ibanez et al. [41,42], the authors show comparable levels of HMW adiponectin in AGA and SGA children at age two; the HMW adiponectin concentrations decrease and become significantly lower in SGA children only at age four.

Interestingly, in this study we found leptin levels to be higher in AGA neonates with respect to SGA infants at birth, in accordance with recent studies [43]. On the contrary, at one year of age SGA infants had leptin levels higher than AGA subjects, although the difference was not statistically significant. Small for gestational age infants show an increase in adipose tissue that is a typical phenomenon after a period of undernutrition. Therefore, we can speculate that the increased levels of leptin may derive from this increased adipose tissue and may be involved in the early development of insulin resistance [28].

Leptin levels of all the neonates at birth were positively correlated with the BMI and head circumference. This may reflect either a simple relationship with adipose tissue or an active role for leptin in foetal growth, since it is known that leptin plays a role in the development of both the foetus and neonate, influencing also head circumference.

In our report we demonstrated comparable insulin levels and HOMA-IR indices between AGA and SGA infants at birth and at 1, 6 and 12 months of age. Only at birth, were levels of insulin in SGA neonates lower than in AGA infants, but the difference was not statistically significant, probably due to the relatively low number of subjects enrolled in the study. However, insulin levels and HOMA-IR index still remained in the normal range in SGA subjects during the first year of age, suggesting that no insulin resistance develops during this period and that insulin alone does not explain changes in adiponectin levels.

The discrepancies between our results and those showing different insulin levels at birth between AGA and SGA infants and a relationship between birth weight with insulin resistance [43-47] could reflect methodological differences in the assessment of insulin sensitivity and insulin resistance.

## Conclusions

In conclusion, minor changes in serum adiponectin, leptin and insulin levels can be observed in AGA and SGA infants during the first year of life. In particular, a progressive decrease in adiponectin levels is observed, while leptin values change according to nutritional status. In fact, leptin levels in all neonates either at birth or at 6 months positively correlated with the BMI (p < 0.05), confirming that leptin is involved in the regulation of body weight and that its plasma level reflects fat reserves.

## Competing interests

The authors declare that they have no competing interests.

## Authors' contributions

EB and AG participated in the design of the study, collection of data and helped to draft the manuscript. CM analysed and interpreted the data, performed the statistical analysis and drafted the manuscript. MA carried out the chromatographic analysis. PT performed the immunoassays. SP was involved in drafting and critically revising the manuscript. MB coordinated the study and helped to draft the manuscript. All the authors have read and approved the final manuscript.
